# How will artificial intelligence and bioinformatics change our understanding of IgA Nephropathy in the next decade?

**DOI:** 10.1007/s00281-021-00847-y

**Published:** 2021-04-09

**Authors:** Roman David Bülow, Daniel Dimitrov, Peter Boor, Julio Saez-Rodriguez

**Affiliations:** 1 University Hospital RWTH Aachen, Institute of Pathology, Aachen, Germany; 2 Faculty of Medicine, Heidelberg University, Heidelberg, Germany; 3 Institute for Computational Biomedicine, Heidelberg University Hospital, Bioquant, Heidelberg, Germany; 4 Department of Nephrology and Immunology, University Hospital RWTH Aachen, Aachen, Germany; 5 Faculty of Medicine, Joint Research Centre for Computational Biomedicine (JRC-COMBINE), 52074, RWTH Aachen University, Aachen, Germany; 6 Molecular Medicine Partnership Unit, European Molecular Biology Laboratory and Heidelberg University, Heidelberg, Germany

**Keywords:** IgA nephropathy, Omics, Artificial intelligence, Imaging, Bioinformatics

## Abstract

IgA nephropathy (IgAN) is the most common glomerulonephritis. It is characterized by the deposition of immune complexes containing immunoglobulin A (IgA) in the kidney’s glomeruli, triggering an inflammatory process. In many patients, the disease has a progressive course, eventually leading to end-stage kidney disease. The current understanding of IgAN’s pathophysiology is incomplete, with the involvement of several potential players, including the mucosal immune system, the complement system, and the microbiome. Dissecting this complex pathophysiology requires an integrated analysis across molecular, cellular, and organ scales. Such data can be obtained by employing emerging technologies, including single-cell sequencing, next-generation sequencing, proteomics, and complex imaging approaches. These techniques generate complex “big data,” requiring advanced computational methods for their analyses and interpretation. Here, we introduce such methods, focusing on the broad areas of bioinformatics and artificial intelligence and discuss how they can advance our understanding of IgAN and ultimately improve patient care. The close integration of advanced experimental and computational technologies with medical and clinical expertise is essential to improve our understanding of human diseases. We argue that IgAN is a paradigmatic disease to demonstrate the value of such a multidisciplinary approach.

## Introduction

IgA nephropathy (IgAN) is the most common primary glomerulonephritis in Europe and especially in Asia. A large proportion of patients develop chronic kidney disease (CKD), with a variable rate of progression, and up to 30% of patients reach end-stage kidney disease (ESKD) within 20–30 years, requiring dialysis or kidney transplant, both of which have a huge economic burden and high mortality [1]. IgAN is designated as an orphan disease (EU/3/16/1778) and apart from nonspecific immunosuppression, which might have considerable side-effects, and supportive therapy, no specific treatments for IgAN currently exist.

Over the years, our understanding of the complex pathology of IgAN has increased significantly [1–3] and led to the so-called 4-hit hypothesis: (i) first, abnormally increased levels of hypo-galactosylated immunoglobulin A1 (Gd-IgA1) are produced, most likely by the mucosal immune system [4], and reach the systemic circulation; (ii) specific auto-antibodies recognize this Gd-IgA1, (iii) this leads to the formation of immune complexes; (iv) finally, these complexes are deposited in the kidney’s glomeruli, leading to chronic inflammation and glomerular injury that lead to the organ's functional decline and failure.

This theory is widely accepted, but there are many open questions, ranging from the origin of Gd-IgA1 to the role of B cells in the pathogenesis of IgAN. There is evidence that mucosal immune response, especially in the gut, is the key source of Gd-IgA1 [4]. To what extent the Gd-IgA1 is released into the blood or instead, B cells migrate to other organs, in particular the bone marrow and tonsils, remains unclear [5]. Other open questions include the role of the various IgA receptors and of the complement system. It also remains open if IgAN is “one disease,” or perhaps several different diseases, and why some patients progress fast while others do not.

While IgAN manifests itself in the kidney, it is a systemic disease in which several organs might be involved, e.g., gut, tonsils, or bone marrow. In some cases, it is also present along with other diseases affecting other organs, especially the liver [6] and the gut [4]. Multiple cell-types in the kidney and other organs are involved, particularly immune cells, and even microbes in the gut [7] and tonsils [8]. The involved molecular processes range from host-pathogen interactions, IgA processing to fibrosis. To improve our understanding of IgAN, and thereby potentially improve patient stratification and treatment, multi-organ, multi-level analyses are required.

Such comprehensive analyses are now possible due to the advances in experimental and computational technologies, characterizing in high-throughput and high comprehensiveness the molecular processes within cells, spanning from the genome to the metabolome [9, 10]. In this review, we introduce the major methodological developments that might advance our understanding, monitoring, and treatment of IgAN. Given the diverse topics, we provide some general concepts and point the reader to more dedicated and detailed reviews. We focus on applications to kidney disease and, whenever possible, highlight examples on IgAN. We finally discuss the potential future applications of these methodologies in IgAN.

## Big data in IgA nephropathy

The term big data has been coined to refer to the increasingly available large amounts of data. In biomedicine, diverse novel technologies have been developed to generate such quantities of data at different scales, from molecules to clinical readouts (Table 1). We here discuss two major areas, molecular “omics” and tissue imaging, although others, such as the data generated by wearables, can be of importance for IgAN [9].

**Table 1:** Outline of big-data methodologies and their applications in “omics” fields

Field	Method	Definition
Genomics	High throughput sequencing	Massively-parallel, rapid, and cost-effective sequencing techniques; also known as next-generation sequencing (NGS)
Transcriptomics	Bulk RNA-Seq	Provides a quantitative snapshot of the expressed transcripts in a pooled sample of cells or tissue; obtained from synthesis of DNA molecules (cDNA), complementary to the transcripts, and their subsequent amplification
	Single-cell RNA-Seq	Enables gene expression quantification at the individual cell level; prior to RNA sequencing, individual cells are sorted or embedded in droplets with specific barcodes
	Spatial transcriptomics	Fluorescent microscopy probes binding to specific transcripts and barcoding methods, targeting synthesis, are used to provide positional context for expressed genes
Proteomics	Targeted proteomics	Mass spectrometry is used to quantify a specific group of known proteins (and/or their modifications)
	Untargeted proteomics	High-throughput mass-spectrometry techniques that aim to quantify the abundance of all proteins within a sample (and/or their modifications) and identify novel ones
Metabolomics	Targeted metabolomics	Quantitative or semi-quantitative approaches in which techniques, such as mass spectrometry and nuclear magnetic resonance spectroscopy, are optimized for a defined set of biochemically-annotated metabolites
	Untargeted metabolomics	Discovery-based approaches that aim to quantify all small molecules within a sample, including novel ones
Microbiome analyses	16S rRNA analysis	Regions of the bacterial 16S ribosomal RNA gene are amplified and used to infer the bacterial taxonomic composition of a sample; high-throughput sequencing of the entire 16S rRNA gene and the denoising of sequence variants have become recently feasible
	Shotgun metagenomics	Uses high-throughput sequencing techniques to characterize the genetic material within a sample, hence enabling the taxonomic composition and functional potential of microorganisms to be inferred; similar methods targeted at transcripts and proteins exist
Imaging	Multi-epitope ligand cartography	Repeated staining, imaging, and bleaching cycles are used to construct toponome maps of tissues/cells
	Exchange—points accumulation for imaging in nanoscale topography	Several antigens can be visualized using fluorescently-labeled oligonucleotides, that bind to antibodies with DNA-PAINT docking sequences, in iterative cycles; the same laser and dye are used for each probe
	Co-detection by indexing	Antibody-binding events are detected using DNA-antibody tags with 5′-overhangs which are sequentially extended by a polymerase incorporating tagged nucleotides in a specific cycle; enables simultaneous cell-resolution imaging of FFPE tissues with at least 66 markers
	Matrix-assisted laser desorption/ionization mass spectrometry imaging	Many analytes can be visualized directly on tissue samples with spatial resolution using their mass-to-charge ratio
	Imaging mass cytometry	Uses isotope-labeled antibodies and mass spectrometry to visualize multiple proteins per FFPE section
	Non-invasive imaging	Radiology and nuclear medicine techniques such as CT, MRI, SPECT, or sonography. Molecular imaging (e.g., Elastin-Imaging) is a new development in kidney fibrosis monitoring.

### Omics data

**Fig. 1:**
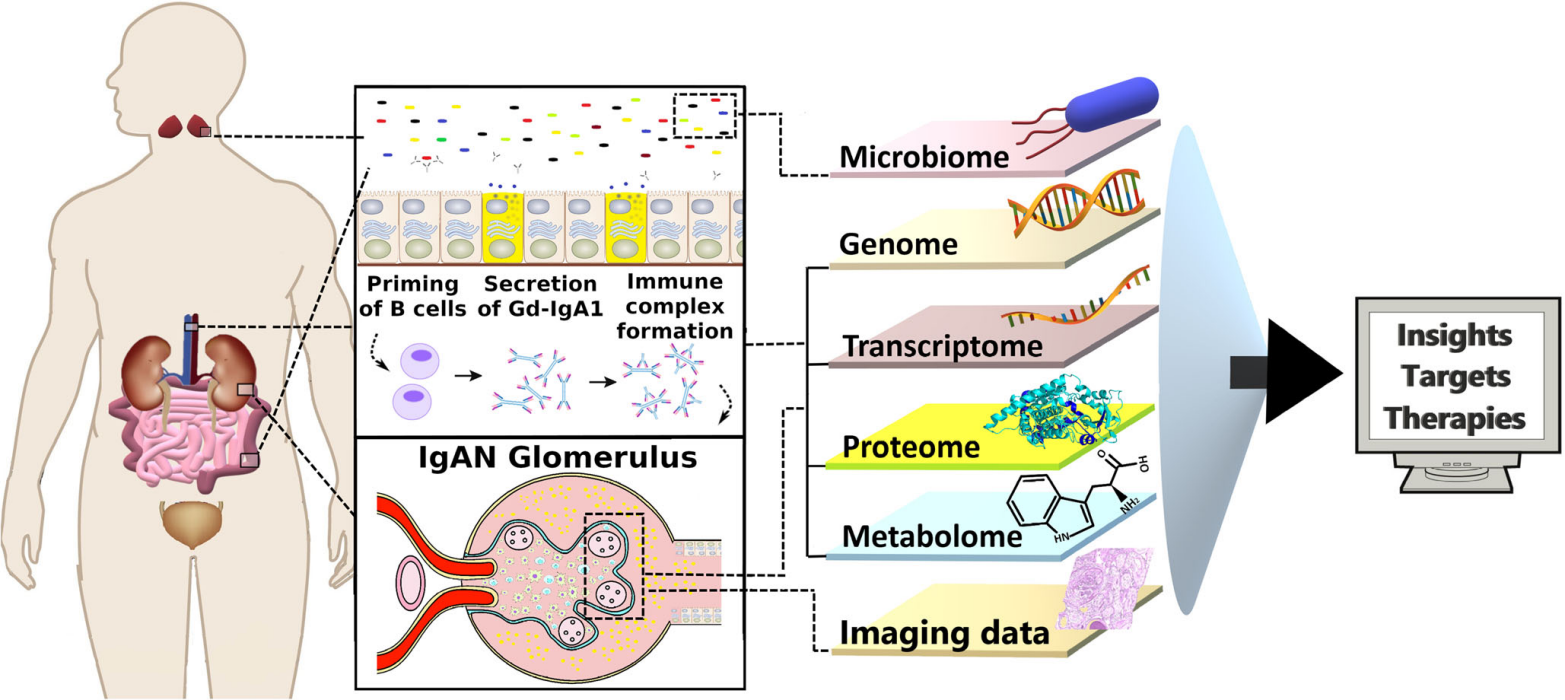
Overview of big-data experimental technologies and how they can improve our understanding of the pathophysiology of IgA nephropathy

Our ability to measure diverse biomolecules at a large scale and speed has increased dramatically over recent years. This spans from DNA (genomics) and RNA (transcriptomics) sequencing to mass spectrometry applied to proteins (proteomics) and metabolites (metabolomics). These methods are collectively called omics and are increasingly able to provide information at the single-cell level and even from tissues, preserving information on the location of the cells. These methods are increasingly being used in nephrology [9–12]. Omics approaches in the kidneys and other organs involved in IgAN can identify novel biomarkers and improve our understanding of the disease mechanism (Fig. 1).

The pathogenic complexity of IgAN is highlighted by its complex genetic basis [13, 14]. Genome-wide association studies (GWAS) have found variations in genes involved in the immune system, including antigen-presentation, alternative pathway of the complement system and mucosal immunity. These findings have provided a genetic basis to the 4-hit theory of IgAN. Collectively, GWAS studies in IgAN have found nearly 20 independent risk alleles, yet these only explain 7% of the disease risk, although it is expected that this will increase with larger cohorts in the future [13].

As an immediate readout of our genome, transcriptome profiling is an attractive strategy to characterize diseases. Due to the relative ease of generating this data, it has been broadly applied in kidney diseases [9, 10], including IgAN. Recent technological developments have made it possible to measure the transcriptome of individual cells, i.e., single-cell RNA sequencing (scRNA-seq). This substantially increases our capacity to examine disease mechanisms [15], including the immune system [11]. scRNA-seq has allowed, for example, to dissect the key cells involved in scar formation in the kidney [16], understand the distribution of distinct immune cell populations in the kidney [17], and identify protective mechanisms mediated by nuclear receptors [18]. A first study applied scRNA-seq to kidney cells and monocytes from peripheral blood of 13 IgAN patients and compared these to 6 controls [19]. The analysis found upregulation of JCHAIN, a gene involved in the dimerization of IgA in mesangial cells, and altered expression profiles of macrophages and CD8+ T-cells that could lead to a deregulation of inflammation. These results illustrate the value of these technologies, but must be taken with caution, given the limited number and the relative heterogeneity of the patients studied.

Besides the commonly measured messenger RNA, other forms of RNA with regulatory roles, such as microRNAs, can be measured with sequencing technologies. A recent study found four microRNAs (-150-5p, -155-5p, -146b-5p, -135a-5p) to be differentially expressed between IgA nephropathy progressors and non-progressors. The most deregulated, miR-150-5p, was found however to be a general meditator of fibrosis rather than specific of IgAN [20].

Messenger RNAs are typically translated into proteins. Although their measurement at large scale, called proteomics, is more challenging to scale up compared to nucleotide-based molecules, it has improved substantially [12]. Besides the expression levels of proteins, their post-translational modifications can be informative, as they can regulate protein function. In IgAN, the aberrant glycosylation of IgA1, that that leads to immune complex deposition and disease pathogenesis, is actively investigated [21]. More generic profiling of blood proteins and peptides can provide biomarkers and molecular signatures. One study analyzed nine published urinary proteomics datasets and integrated them with transcriptomic data and literature knowledge to identify twenty proteins involved in IgAN in the kidney [22]. The relevance of three of these proteins (adenylcyclase–associated protein 1 (CAP1), SHC-transforming protein 1 (SHC1), and prolylcarboxypeptidase (PRCP)) was experimentally confirmed [22]. Finally, proteomics, like transcriptomics, is becoming increasingly available with spatial resolution from tissues [12], paving the way to generate multiplexed-histological data to complement classic pathological assessment.

Metabolomics analyses provide a snapshot of the metabolites within samples. This snapshot serves as a metabolic signature of the cellular processes driven by the transcriptome and proteome. Metabolomics approaches have been extensively applied in CKD to identify metabolic changes, biomarkers, and signatures [23]. In an early retrospective metabolomics analysis, 16 plasma metabolites were associated with CKD incidence in a follow-up period of 8 years. Five of these, i.e., 5-hydroxyindoleacetic acid, citrulline, kynurenic acid, kynurenine, and xanthosine), were identified as eGFR-independent CKD predictors and were used to improve the predictive ability of a logistic regression model with clinical risk factors, such as proteinuria and eGFR [24]. Another profiling of the plasma metabolome reported 16 metabolites as possible predictive risk markers for primary outcomes of progression to ESKD and death in a longitudinal cohort [25]. Metabolic alterations in the urine [26–28] and fecal samples [26] of IgAN patients when compared to healthy controls were also described. These studies made attempts to associate changes in free amino acids and p-cresyl levels with disease progression [26], aromatic amino acid metabolism and biosynthesis with disease severity [27], and betaine and citrate with regulation of the inflammatory marker TNF-α [28]. These results are encouraging but the reported metabolic changes often overlap with alterations described in general CKD, such as p-cresyl and derivatives of tryptophan metabolism. Larger cohorts and follow-up studies are needed to characterize and confirm these metabolic alterations in IgAN and their specificity for the disease.

The human microbiome encompasses the microbial communities that occupy distinct parts of the human body such as the skin, tonsils, and gut. Recent advances in high-throughput technologies have substantially expanded our understanding of the microbiome complexity and dynamics, as well as its alterations in various diseases, including CKD [29] and inflammatory bowel disease (IBD) [30].

A sequencing-based technique targeting the bacterial 16S ribosomal RNA gene has been widely used to profile the taxonomic composition of the microbiome in different conditions. 16S analyses in IgAN reported alterations in the microbiome’s structure in the saliva [31], tonsil swabs [32], and gut [33–35], while denaturing gradient gel electrophoresis showed compositional changes in tonsil tissues [36]. These taxonomic alterations were noted in IgAN patients when compared to healthy controls [26, 31, 32, 34, 36] or other nephropathy patients [32, 33]. Some of the analyses also linked specific microbiota with remission rates [26, 36] and clinical measurements, such as proteinuria [34], serum albumin [32, 33], and inflammatory markers [34], thus highlighting the microbiome’s potential as a diagnostic and prognostic marker of IgAN.

The role of bacteria as potential inductors of IgAN pathology is further supported by their association with exaggerated antibody production in IgAN patients when compared to controls [37, 38] and deposition of IgA antibodies and bacterial antigens in the glomeruli [39–41]. Mice overexpressing B cell–activating factor (BAFF) showed that the presence of microbiota was essential for the development of a phenotype mimicking IgAN pathophysiology [42]. Moreover, IgAN phenotype was delayed or prevented in mice expressing a human IgA1 variant prone to mesangial deposition, when grown under germ-free conditions or upon antibiotic-induced microbiome depletion, respectively [43, 44]. Recently, binding of polymeric IgA (pIgA) to certain microbiota was found to be enriched in the tonsil crypts of IgAN patients and IgA binding intensity to the same taxa correlated with Gd-IgA1 serum levels [8]. Yet, a preceding analysis reported no significant alterations between the tonsillar microbiome of IgAN and recurring tonsillitis patients [45]. These data suggest that an excessive mucosal immune response [46] against particular taxa might underlie glomerular immune-complex deposition in IgAN [8].

Albeit promising, IgAN human microbiome analyses were performed on small cohorts of ethnically uniform patients, and data on key confounders are missing, such as the use of immunosuppressants. Furthermore, more in-depth techniques, such as Shotgun Metagenomics, which attempts to quantify all genetic material within a sample, can be used to provide higher taxonomic resolution and pinpoint the metabolic or functional changes in the IgAN microbiome.

### Imaging technologies

Several techniques enable the analyses of molecular expression patterns directly on tissue sections. Such techniques can be especially interesting for analyses of rare tissues, such as kidney biopsies. We discuss some examples that were also used in nephrology and nephropathology, acknowledging that this is not comprehensive and represents only selected methods.

Multiplexing techniques enable visualization of multiple molecular targets at once, providing an advantage compared to traditional immunofluorescence techniques, which are usually limited to 4–5 markers (colors). Multi-epitope ligand cartography (MELC) is a high-throughput immunofluorescence method that relies on repeated cycles of staining and bleaching, enabling to compile a so-called toponome map, i.e., the expression of target molecules in a cell or tissue [47]. Theoretically, this approach can be used to visualize expression of any molecule to which a fluorescently labelled ligand is available.

Another technique, the points accumulation for imaging in nanoscale topography (PAINT) [48, 49], also enables high resolution tissue multiplex analyses [50]. Exchange-PAINT uses fluorescently labeled oligonucleotides that bind to antibodies tagged with a DNA-PAINT docking sequence. To visualize several antigens, iterative cycles consisting of staining, imaging, applying a unique pseudocolor, and washing are performed. Importantly, Exchange-PAINT can be performed using a single dye and laser, allowing to choose the dye with optimal intrinsic properties for the imaging tasks for all probes [50].

Co-detection by indexing (CODEX) uses dyed nucleotides for multiplex tissue analysis. CODEX uses DNA-antibody-tags with specific 5′-overhangs that are sequentially extended by a polymerase in each cycle. This way in each cycle only tags of defined antibodies will incorporate the dyed nucleotides. After incorporation, imaging is performed and the dyed nucleotides are removed by inter-cycle Tris(2-carboxyethyl)phosphine hydrochloride (TCEP) cleavage [51]. This enables simultaneous imaging of 66 markers in formalin-fixed and paraffin-embedded (FFPE) tissue [51]. Theoretically, the analyses can be performed using a standard immunofluorescence microscope.

A similar technology, imaging mass cytometry (IMC), can be used to visualize multiple proteins in FFPE sections at once and has recently been applied to kidney tissue [52]. IMC uses special antibodies that are conjugated to specific isotopes. The tissue is meandered using a laser with a resolution of 1 μm, aerosolizing, atomizing, and ionizing it. Then, the tissue is fed into a mass spectrometer for isotope abundance analysis, which identifies the respective antibodies at a given location, providing spatial expression information. For visualization, the final image must be constructed computationally. A recent study applied IMC to human kidneys and found a potentially novel cell type in the distal convoluted tubule (DCT) that does not express calbindin (a typical DCT-marker) and is larger than an intercalated cell [52].

Matrix-assisted laser desorption/ionization mass spectrometry imaging (MALDI-MSI) can analyze many analytes directly on tissue samples with reasonable spatial resolution. A molecule of interest can be identified using the mass-to-charge ratio (m/z). This technique was recently applied to IgAN [53]. By comparing eleven IgAN cases to six non-IgAN cases with a mesangioproliferative glomerular injury pattern, the authors could identify proteomic signatures associated with progressive IgAN, e.g., increased glomerular vimentin expression [53].

The methods above were largely applied to 2D tissue sections. 3D tissue imaging represents an interesting alternative with some advantages over 2D section imaging, particularly for the assessment of structures like vessels or glomeruli. Such 3D tissue imaging can be destructive, i.e., when the tissue needs to be fully processed for the method making it unavailable for further analyses, or non-destructive; i.e., the tissue remains available and can be used for other “destructive” molecular methods. MicroCT imaging of tissues is one example of non-destructive imaging that has already been used in kidneys [54]. Optical tissue clearing is another interesting approach for 3D organ visualization, feasibility of which has already been shown in the kidney [55].

Finally, all non-invasive imaging methods of radiology and nuclear medicine, i.e., sonography, computed tomography (CT), magnetic resonance imaging (MRI), positron emission tomography (PET), and single-photon emission computed tomography (SPECT), provide spatial and non-invasive morphological information. There are substantial developments in each of these imaging modalities, including technological developments, such as super-resolution sonography, or various specific MRI imaging sequences and techniques. Another interesting development is the non-invasive molecular imaging of kidney diseases, as recently shown for imaging of fibrosis [56, 57]. Given that all these techniques provide images, AI approaches are increasingly being developed and implemented for augmented diagnostics and analysis.

## Introduction to artificial intelligence and bioinformatics

The technologies summarized in the previous section generate large amounts of data. To extract knowledge from this data, advanced computational methods are required. The analysis of biological data has been historically the focus of the field of bioinformatics. This field utilizes the combination of expertise in biology, computer science, statistics, and other fields to develop software and methods to process, store, and analyze large data. The analysis has been typically based on methods from statistics and artificial intelligence (AI). In recent years, the field has witnessed a dramatic advancement thanks to new developments in AI, with profound implications particularly for pathology.

### Artificial intelligence, machine, and deep learning

Various definitions of artificial intelligence (AI) exist, e.g., John McCarthy, one of the founding fathers of AI, defined AI as follows: “AI is the science and art of making intelligent machines” [58]. Machine learning (ML) is a subdiscipline of AI concerned with building systems that can learn representative patterns from data. Deep learning (DL) is a subdiscipline of ML making use of artificial neural networks (ANNs) [59].

There are several different types of neural networks, e.g., recurrent neural networks that are primarily suited for sequence data, or convolutional neural networks (CNNs) that are primarily suited for image data. In medicine, there has been considerable interest in DL-based processing of image data, especially in radiology and pathology [60–65]. Training of ML and DL algorithms can be supervised, semi-supervised, or unsupervised. Supervised training uses datasets consisting of data (e.g., images) and labels (e.g., disease classification like IgA-nephropathy, lupus nephritis, or outcomes such as disease progressor vs. non-progressor or treatment-responders vs. non-responders). In this scenario, each image has a label that in most cases must be provided (“annotated”) by an expert. This is labor-intensive and might limit generation of large-scale datasets.

Unsupervised learning does not use labels. Instead, the unlabeled training data is grouped based on automatically recognized similarities. This allows us to find previously unknown patterns in data. A medical application could be to group patients suffering from a multifactorial disease based on clinical and molecular data. In semi-supervised learning, some data has labels, and some data does not. The goal is to thereby expand the training data, when a dataset consists of large amounts of unlabeled data and some labeled data.

Reinforcement learning is different from supervised and unsupervised learning. The key difference to supervised learning is that there is no labeling, but an algorithm acts in a specific environment to maximize a defined reward. This type of machine learning was successfully implemented in games such as Go [66] or Starcraft [67], but currently is only rarely applied in medicine. Still, some applications have been described, e.g., the AI clinician that can suggest optimal treatments for adult sepsis patients [68]. However, the “reward” might be difficult to define in medicine and might change during disease, e.g., when transitioning from a curative to a palliative therapeutic approach in a cancer patient.

Correctly labeled ground truth is critical for effective supervised ML and DL development. If the ground truth is false or biased, model evaluation and performance will be unreliable. Currently, there are no means or techniques to evaluate the necessary amount of data for “successful” model development a priori, as is the case in clinical trials when calculating the required sample size. In general, deep learning performs better with increasing amounts of data [69], although this amount can vary substantially for different approaches. E.g., already a couple of annotated glomeruli can suffice to train a DL algorithm to detect them with high accuracy [70, 71], while many thousands of annotations are required for the reliable detection of peritubular capillaries [72].

In medical applications, most studies in ML and DL use the terms training dataset, testing dataset, and validation dataset. The training dataset is used for model development, and the testing dataset is used for performance evaluation. Training and testing datasets are often compiled from data of one center, but ideally should be from multiple centers. The validation dataset is used to determine a model’s generalization capability, i.e., the ability to perform the respective task on previously unseen data. If trained only on one cohort, a model might over-fit to the characteristics specific only to this cohort, i.e., essentially learn the data by heart. In such a case, performance can seem very high, but the model will fail on an external “independent” dataset. For robust evaluation of model performance, it is vital to use an external validation dataset.

## AI applications for medicine

The amount of healthcare data is expected to rise from 153 Exabytes (i.e., 153 billion Gigabytes) in 2013 to 2314 Exabytes in 2020 [73]. Not only the amount increases but also the data are becoming more complex, having multiple dimensions. For example, semantic, numerical, and image data that include basic characteristics (such as age), medical history (such as previous diseases), results from multiple medical curative or diagnostic interventions, radiology and pathology image data, laboratory data, and genetic and other omics’ data. The main reasons for the increase in medical data are the digitalization of medicine (e.g., through electronic health records), omics approaches (such as next-generation sequencing and especially single-cell sequencing), and digitalization of image-based disciplines such as radiology and pathology.

In the following, we will focus on machine and deep learning in nephrology and nephropathology. More general overviews on machine and deep learning in medicine and omics can be found elsewhere [60, 74–76]

### AI applications for nephrology

The use of ML and DL applications in nephrology is still in its infancy [77]. Most studies have focused on acute kidney injury (AKI) to enable earlier detection [78–81].

An AKI alert system was recently developed using a recurrent neural network that continuously monitors electronic health records. The model could predict future AKI of any severity with an area under the receiver operating characteristic curve (AUROC^1^) of 0.921 and an area under the precision-recall curve (AUPRC^2^) of 0.297 up to 48 h in advance [79]. 90.2% of all AKI episodes that required dialysis were correctly identified. Although this accuracy is very promising, future prospective validation will be needed to assess the true impact on patient care. One limitation of this study is that the model was developed on a US Department of Veterans Affairs dataset that e.g. contained only 6.38% female patients, with lower model performance in women [79]. This example illustrates the need for transparency in the data used for model training and development.

In a comparative study, “Streams” by “Google Health,” a commercial AI-powered app that can warn when AKI is about to occur, was implemented in one center (The Royal Free Hospital, RFH, London) and clinical outcome was compared with another center (The Barnet General Hospital, BGH, London) [82]. By using this app, time to AKI prediction and nephrotoxicity treatment significantly improved. However, there was no difference in renal recovery rate, the primary outcome of the study, after implementation of the app.

The AKIpredictor tool [80] is a ML-based tool for the prediction of AKI in critically ill patients. It has recently been prospectively validated within the scope of a clinical trial (NCT03574896) and showed similar discriminative performance as physicians [81]. However, this trial was performed in a single center and physicians had three additional hours to make their predictions, having access to more information than the algorithm, which might decrease the grade of evidence.

A system based on recurrent neural networks was developed for real-time prediction of severe complications after cardiac surgery based on 9269 patients and validated on an external dataset of 5898 patients [83]. This system had a positive predictive value of 0.87 and a sensitivity of 0.94 for prediction of AKI requiring dialysis. Importantly, this system works with routinely collected clinical data without the need for manual intervention. Since it works in real time, the application of such a system could go beyond simple prediction and potentially be used to assess treatment response as well [83].

There has been considerable effort to test ML in kidney transplantation [84–87]. A ML classifier system was developed based on the molecular profiles of 1208 kidney transplant biopsies from 13 international centers. The output of the system was a score for six archetypes of rejection: no rejection, T cell–mediated rejection (TCMR), three different archetypes for antibody-mediated rejection (ABMR) (early-stage, fully developed, late-stage), and mixed rejection [84]. One of the advantages of such a system is that each classification is assigned a level of confidence, providing an assembly of probabilities of the defined archetypes for each case. However, there was considerable disagreement with the histologic assessment of the biopsies (in total 32%, for some diagnoses up to 94%) [84], explained by the authors mainly due to inconsistencies between pathologists and problems in the Banff-classification for kidney allograft pathology. This system was recently further improved (Molecular Microscope Diagnostic System, MMDx) showing slightly more agreement with histologic diagnoses of 78% for TCMR and 73% for ABMR (balanced accuracies) [85]. A prospective investigation of this new approach within the framework of a clinical trial would be highly interesting.

ML is also starting to be applied to omics data in the kidney [9, 88]. The increasing availability of such data, in particular via large consortia like the Kidney Precision Medicine Project (KPMP), opens the door to apply these methods in nephrology [9, 10], with expected increase in the coming years.

### AI applications for nephropathology

Pathology, including nephropathology, is expected to strongly benefit from the advances in computer vision, especially through DL. While there have been several studies on DL in oncologic pathology [61, 65, 89–91], often outperforming human pathologists (e.g., detection of genetic alterations from histology images alone), the use of DL in nephropathology is only starting to emerge [92].

Much of AI research in nephropathology is currently concerned with semantic segmentation, i.e., breaking down an image into specific parts and assigning a label to each pixel (e.g., glomerulus). Detection and segmentation of glomeruli in digital pictures of histological specimens or whole slide images (WSI) was one of the first and commonly used tasks, shown to be feasible in multiple stains [70, 93–95]. More recently, semantic multiclass segmentation of kidney histology was developed by several groups [72, 96, 97].

The first study in this area developed a CNN for semantic segmentation of kidney histology into multiple compartments in periodic acid Schiff (PAS)–stained human kidney allograft specimens. The segmentation classes also included atrophic tubules and sclerotic glomeruli. This enabled automatic quantification of the number and percentage of globally sclerotic glomeruli, which is a standard readout of kidney biopsy diagnostics and assessment of prognosis. A high correlation (spearman correlation coefficient of 0.81) was described between the CNNs measurement of fibrosis and the estimates of two pathologists [96].

The feasibility of multiclass segmentation in various stains commonly used in nephropathology diagnostics, i.e., Hematoxylin & Eosin (H&E), PAS, Jones-Silver, and trichrome using CNNs, was investigated in another study. These CNNs were developed on a large multicenter dataset of minimal change nephropathy biopsies, the NEPTUNE dataset [72, 98]. Currently, this is the only study showing feasibility of kidney capillary segmentation, which required an enormous training effort. Segmentation of capillaries could prove especially useful in kidney allograft pathology since inflammation of peritubular capillaries is a quantitative canonical lesion of ABMR. Additionally, optimal magnifications for the segmentation of different histological compartments were investigated [72].

Another study developed a CNN for multiclass segmentation in experimental nephropathology. This CNN can perform semantic segmentation in multiple murine models of kidney diseases, as well as healthy kidneys from multiple species including humans. This approach enabled quantitative measurements of the segmented histological compartments, enabling high-throughput reproducible quantitative analysis of kidney histology that showed good correlation with other standard measurements [97].

An automated computational pipeline for analysis of glomeruli from patients with diabetic nephropathy in WSI was also described [99], which detects glomeruli, identifies and discretizes glomerular components, quantifies them, and finally classifies glomerular features. Sequences of the glomerular features were fed into a recurrent neural network that provided the final output of classification of diabetic glomerulopathy. By systematically dropping out specific features, their respective impact for classification was determined [99]. Although not yet available, similar approaches can be envisioned for the analysis of IgA nephropathy or prediction of clinical parameters directly from histology in the future.

**Fig. 2:**
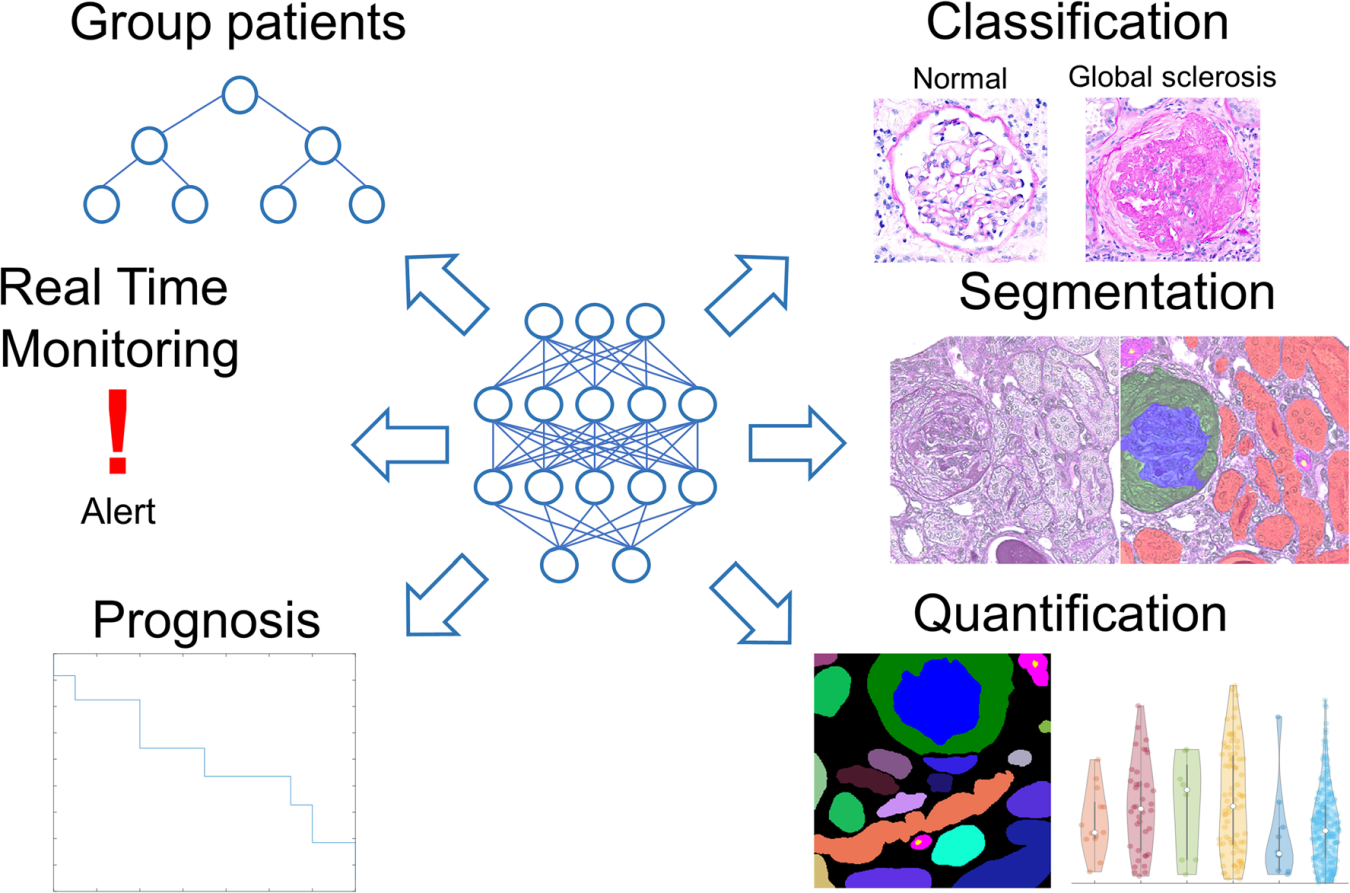
Examples of AI-based applications for nephrology and nephropathology. AI-based methods have been primarily applied to nephrology to group patients based on outcome, perform real-time monitoring of acute kidney injury or to establish a prognosis. In nephropathology, main applications include classification (mostly of glomeruli) and semantic segmentation, often combined with quantification of the segmented compartments

For all these models to be applicable on an international scale and in clinical trials, consensus definitions for histological compartments, as well as for histological lesions as have been recently published for glomeruli [100], need to be considered during development (i.e., defining the “ground truth”). Examples of applications for AI in nephrology and nephropathology are given in Fig. 2.

### AI applications for IgA nephropathy

There is only scarce literature on ML or DL applied to IgAN. The available machine learning studies on IgAN focus on the prediction of clinical outcomes with the goal of establishing a prognosis for individual patients.

An early study from 1998 used an ANN with only a single hidden layer to identify IgAN-patients with poor prognosis and model predictions were compared to predictions of six nephrologists [101]. The ANN showed a sensitivity of 86.4% for identification of patients with progression of disease, which was superior to the mean sensitivity of 72% in the group of nephrologists. However, only a small training dataset of 54 patients was used, and no external validation was performed.

One study analyzed the performance of several different machine learning models to predict the development of ESKD in IgAN patients [102]. A large cohort of 1174 patients was used to train the models. A neural network performed with the highest accuracy (more than 90%) for prediction of ESKD and was implemented as a web-based decision support system [103].

Recently, another ML model for IgAN was built to predict whether a given patient will develop ESKD or a decline in glomerular filtration rate within 5 years [104]. The variables with the highest importance were interstitial fibrosis and tubular atrophy (IFTA), serum albumin, and the percentage of globally sclerotic glomeruli [104] highlighting the importance of exact and reproducible quantification of kidney biopsy features [96, 97].

Another study investigated the possibility to identify IgAN patients responsive to immunosuppressive therapy using data from 4047 patients across 24 centers in China [105]. The primary readout used to define the benefit of immunosuppressive treatment was time to ESKD. The authors deployed a model-based recursive partitioning, a machine learning method to computationally group patients based on clinical and pathological variables, so that the treatment response is similar within each final group, but different between the final groups. Thus, the method identified patient groups showing a good response to immunosuppressive therapy and patient groups showing a bad response. This allowed to create a partitioning tree, in which each node is split based on a clinical characteristic (e.g., serum creatinine (SCr), diastolic blood pressure). Such a tree can be used to classify individual patients. Based on this analysis, the authors found that IgAN patients with a SCr < 1.437mg/dl and high proteinuria might potentially benefit from immunosuppressive therapy and that IgAN patients with a SCr > 1.437mg/dl, high proteinuria, and crescents in the biopsy have significant benefits of immunosuppressive therapy [105].

Recently, prediction of development of ESKD and prediction of ESKD over time in IgAN patients were achieved using two models, a classifier (that outputs categorical labels, e.g., ESKD) and a regression model (that outputs continuous values, e.g., 1.32 years), implemented into a clinical decision support system that can also run on a mobile phone [106]. The classifier model performed with high accuracy (0.92 sensitivity and 0.83 precision) for prediction of ESKD after 5 years and similarly for prediction of ESKD after 10 years. The regression model that predicted the time-point of ESKD development had a mean absolute error of 1.78 years. The prediction tool was evaluated on an external cohort consisting of 167 patients from six kidney units with a prediction error of only 8.4% (it did not predict six ESKD occurrences and wrongfully predicted ESKD in eight cases out of 167) [106].

## Conclusions

The digital and big data transformation of medicine is ongoing. Especially image-based diagnostic disciplines such as radiology or pathology, but also clinical disciplines, will experience changes in the way they work [60]. Electronic health records, omics approaches, and emerging imaging technologies can provide large-scale multi-dimensional data on IgAN patients.

The availability and integration of these will greatly enhance the ability of physicians to formulate a diagnosis and prognosis or decide on treatment and management of patients. However, with the growing complexity and amounts of data, this will become increasingly challenging for humans and will not be possible for physicians alone. Humans can make complex assumptions based on few data points but are overwhelmed by massive amounts of data. The opposite is true for machines. ML and DL in medicine can be used as diagnostic or data mining tools, e.g., to guide therapy and provide predictive data guiding patient management. ML and DL can also be used to investigate complex relationships, such as treatment responses without the need of prior hypotheses. The synergy between humans and “intelligent” machines can potentially further accelerate and improve personalized precision medicine. The combination of human and artificial intelligence is referred to as augmented intelligence and will likely prove the best way forward [107].

There are various hurdles that need to be addressed to facilitate this transformation. Most studies for DL and ML in healthcare are still retrospective and lack the level of evidence needed before their clinical applicability. The performance of ML /DL techniques might differ in a retrospective setting when compared to “real-world-data.” Additionally, when compared to experienced physicians, there might be differences in physician performance in a retrospective setting and a true clinical setting. Moreover, well-annotated large datasets, ideally coming from multiple international centers, are crucial for robust development of deep learning techniques. However, in nephrology, such datasets are largely missing [92]. Sample sizes in omics studies need to be increased to provide more reliable read-outs and confounding factors, e.g., influence of diet in studying microbiome or urine, and need to be addressed systematically. Likely, sufficient group sizes will not be achievable by a single research group, but need extensive, best international collaborations. This is particularly important for IgAN, which has a relatively low prevalence. Furthermore, privacy concerns must be considered when collecting datasets that include patient data, especially when they are transferred to private companies [108]. Methods such as federated learning, combined with high standards for encryption, might be a possible way to facilitate international collaboration in ML and DL projects [109, 110]. For unbiased assessment of the ML methods, crowdsourced open challenges, where any team worldwide can compete to solve a given task in the best way, can be leveraged [111].

There are currently only few studies concerning ML and DL for IgAN. Due to the potential of these emerging technologies, we expect a substantial increase in studies assessing their potential in the coming years. There have been some notable advances predicting disease progression [106, 112, 113], but there is still room for improvement.

ML and DL augmented computer vision applications might further improve pathology diagnostics in IgAN and potentially provide more reproducible quantitative data. Such specific extraction of histological features can lead to more precise and more granular classification systems. Digital oncologic pathology showed that DL can uncover previously unrecognized information contained in histology images, e.g., data on mutations [61] or survival [91]. However, caution is important, since ML models primarily establish correlations and are unable to perform causal inference, which remains an area of active methodological development in AI.

There is growing evidence of the involvement of multiple organs and the immune system in IgAN pathophysiology. A multi-organ systematic analysis of IgAN, particularly incorporating different omics levels, spatial context [114], the microbiome [7], and histology [115] will likely shed light on the open questions around the pathophysiology of IgAN. This will ultimately improve treatment and, e.g., might stratify which patients would eventually benefit from systemic immunosuppression [116], gut-targeted immunosuppression [5] particularly against supportive care eventually combined with new drugs like the sodium-glucose transport protein 2 (SGLT2) inhibitors [117]. In summary, we believe that the application of bioinformatics and artificial intelligence, although there is still a long way to go, will enable personalized precision medicine in IgAN.

## Footnotes

1 The most common performance metrics for classification problems are the AUROC and the AUPRC. The ROC plots the true positive rate on the *Y*-axis against the false-positive rate on the *X*-axis, and the AUROC is the area under the curve that joins the resulting points. AUROC values of 0.5 describe a random classifier and 1.0 a perfect classifier.

2 The PRC plots the precision on the *Y*-axis and the recall (i.e., the true positive rate) on the *X*-axis. AUPRC =1 corresponds to a perfect classifier but the value describing a random classifier depends on the frequency of the event of interest. If an event occurs with 10% probability in a dataset, AUPRC = 0.1 would be random. This makes the AUPRC considerably more difficult to interpret and is likely one reason why this metric is less used than the AUROC in medical deep learning publications, even if the AUPRC is more robust when the number of positive and negative cases is highly unbalanced.
