# Correction to: How will artificial intelligence and bioinformatics change our understanding of IgA Nephropathy in the next decade?

**DOI:** 10.1007/s00281-021-00858-9

**Published:** 2021-05-07

**Authors:** Roman David Bülow, Daniel Dimitrov, Peter Boor, Julio Saez-Rodriguez

**Affiliations:** 1 University Hospital RWTH Aachen, Institute of Pathology, Aachen, Germany; 2 Faculty of Medicine, Heidelberg University, Heidelberg, Germany; 3 Institute for Computational Biomedicine, Heidelberg University Hospital, Bioquant, Heidelberg, Germany; 4 Department of Nephrology and Immunology, University Hospital RWTH Aachen, Aachen, Germany; 5 Faculty of Medicine, Joint Research Centre for Computational Biomedicine (JRC-COMBINE), 52074, RWTH Aachen University, Aachen, Germany; 6 Molecular Medicine Partnership Unit, European Molecular Biology Laboratory and Heidelberg University, Heidelberg, Germany

**Correction to: Seminars in Immunopathology**

10.1007/s00281-021-00847-y

The original version of this article unfortunately contained a mistake. The title was incorrect. The corrected title is given above.

The original article has been corrected.

